# Clinical Effectiveness and Cost-Effectiveness of Pulmonary Rehabilitation for People in Uganda With Post-TB Lung Disease: A Randomized Controlled Trial

**DOI:** 10.1016/j.chest.2025.12.057

**Published:** 2026-03-06

**Authors:** Winceslaus Katagira, Mark W. Orme, Richard Kasiita, Jesse A. Matheson, Matthew Richardson, Andy Barton, Jalia Nanyonga, Michael C. Steiner, Bruce Kirenga, Sally J. Singh

**Affiliations:** a Makerere University Lung Institute, Kampala, Uganda; b Department of Respiratory Sciences, University of Leicester, Leicester, England; c Centre for Exercise and Rehabilitation Science (CERS), NIHR Leicester Biomedical Research Centre–Respiratory, University Hospitals of Leicester NHS Trust, Leicester, England; d Department of Physiotherapy, Mulago Hospital, Kampala, Uganda; e Department of Economics, University of Sheffield, Sheffield, England

**Keywords:** Africa, chronic respiratory disease, education, exercise, nondrug treatment, post-TB lung disease, pulmonary rehabilitation, quality of life, TB

## Abstract

**Background:**

Post-TB lung disease (PTLD) causes significant disability in survivors of TB. Pulmonary rehabilitation (PR) may offer effective disease management but lacks high-quality evidence in this underrepresented population.

**Research Question:**

Compared with usual care, does a 6-week PR program improve exercise capacity and health-related quality of life (HRQoL) cost-effectively in adults living with post-TB lung disease?

**Study Design and Methods:**

We conducted a single-center randomized controlled trial with blinded outcome assessments, comparing PR vs usual care (UC) for adults in Kampala, Uganda with PTLD. Participants were randomized (1:1) to receive either PR or UC, with assessments at 6 weeks postintervention. The primary outcome was change in exercise capacity measured by the Incremental Shuttle Walk Test. Secondary outcomes included HRQoL, respiratory symptoms, psychological well-being, and cost-benefit analysis. A generalized linear mixed model was used for the primary efficacy analysis (intention-to-treat) and a difference-in-differences analysis for secondary outcomes (modified intention-to-treat).

**Results:**

Between November 2020 and September 2022, 178 adults with PTLD were assessed for eligibility and 114 were randomized (mean age ± SD, 43.3 ± 15.2 years; 65 [57%] were male). The postintervention improvement in mean Incremental Shuttle Walk Test in the PR group was significantly greater than in the UC group, by 54.36 m (95% CI, 17.22-91.51 m; *P* = .004). We also observed significant improvements in HRQoL in PR compared with UC: COPD Assessment Test score, –3.6 (95% CI, –6.7 to –0.39; *P* = .015); and Clinical COPD Questionnaire total, –0.37 (95% CI, –0.68 to –0.06; *P* = .004). The EuroQol Visual Analog Scale and quality-adjusted life-years (QALYs) marginally improved in the PR group vs the UC group: 3.98 (95% CI, –2.05 to 10.02; *P* = .191) and 0.02 (95% CI, –0.02 to 0.05; *P* = .334), respectively. The average cost of PR was $6,468/QALY gained, equating to $20,000/QALY gained after adjusting for purchasing power (below the National Institute for Health and Care Excellence cost-effectiveness threshold).

**Interpretation:**

In adults with PTLD, a 6-week PR program elicited clinically and statistically significant improvements in exercise capacity and HRQoL compared with UC, and was cost-effective.

**Clinical Trial Registration:**

ISRCTN Clinical Trial Registry; No.: ISRCTN18256843; URL: isrctn.com

FOR EDITORIAL COMMENT, SEE PAGE 6

**Take-Home Points**

**Research Question:** Compared with usual care, does a 6-week pulmonary rehabilitation program improve exercise capacity and health-related quality of life cost-effectively in adults living with post-TB lung disease?

**Results:** The 6-week pulmonary rehabilitation program demonstrated statistically significant and clinically meaningful improvements in exercise capacity and health-related quality of life compared with usual care, and was cost-effective.

**Interpretation:** In people with disability associated with post-TB lung disease, pulmonary rehabilitation was shown to improve exercise capacity and health-related quality of life, and provide good value for money.

Despite TB being the leading cause of infectious disease deaths, national TB programs have made progress, with 155 million survivors by 2020, including 25.7 million in Africa.^1^ Many survivors of pulmonary TB continue to experience chronic respiratory symptoms after microbiologic cure, partly attributable to the permanent lung parenchymal damage caused by *Mycobacterium tuberculosis*.^2^ Pulmonary TB can cause lasting damage to the airways, lung parenchyma, pleura, or pulmonary vasculature,^3^^,^^4^ leading to persistent respiratory symptoms, a condition known as post-TB lung disease (PTLD),^5^ a type of chronic respiratory disease (CRD).^6^

Adults with PTLD develop secondary systemic impairments including skeletal muscle dysfunction and wasting, systemic inflammation, and oxidative stress.^6^, ^7^, ^8^ The systemic impairments are often compounded by physical inactivity, leading to significant impairment in daily functioning, inability to work, and reduced health-related quality of life (HRQoL).^9^^,^^10^ Considering that most survivors of pulmonary TB are young people, the economic impact on this group can be substantial.^1^ At present, there are no known effective medicines for PTLD. Pulmonary rehabilitation (PR) is a low-cost, high-impact nonpharmacologic intervention that reverses the disability from CRDs such as COPD, and is backed by strong evidence in high-income countries.^11^^,^^12^ However, its efficacy in PTLD-associated disability lacks strong evidence.

Consensus statements on the management of PTLD recommend PR, but, to date, this is not backed by high-quality evidence.^13^ The World Health Organization (WHO) Rehabilitation 2030 initiative calls for robust evidence on rehabilitation for many chronic diseases.^14^ However, data on the clinical efficacy and cost-effectiveness of interventions that reverse PTLD-associated disability remain limited. In a systematic review of interventions to prevent post-TB sequelae, only 6 of 25 studies addressed pulmonary sequelae,^15^ and the PR studies in low-resource, high-TB burden settings were underpowered.^16^

Accordingly, we conducted an adequately powered randomized controlled trial (RCT) with blinded outcome measures to compare PR with usual care in people with PTLD, with the primary outcome as exercise capacity.

## Study Design and Methods

A single-center RCT with blinded outcome assessments comparing PR vs usual care for adults living in Kampala, Uganda with PTLD was conducted.

Consenting participants were randomized 1:1 to receive either PR or usual care (waiting list), using a secure, password-protected web-based system,^17^ which generated a random allocation sequence. An independent team member, using a completed checklist, conducted randomization and relayed study ID and allocation to the data collection and intervention teams, which then informed the participants.

As PR precludes blinding, participants were requested not to disclose their allocation. Blinded staff conducted outcome assessments, prioritizing the Incremental Shuttle Walk Test (ISWT) to minimize unblinding risk, and followed standardized procedures requiring documentation of any suspected unblinding.

The study was conducted at the PR center of the Lung Institute Clinic, an academic outpatient facility with a skilled PR team of health care professionals delivering PR to patients with PTLD and COPD for nearly 8 years before the study. Adults with PTLD were referred from TB and HIV/TB care centers. PTLD was defined as completion of treatment for microbiologically confirmed pulmonary TB, with persistent respiratory symptoms at enrollment, and radiologic evidence of lung parenchymal damage consistent with TB on frontal chest radiography, confirmed by a radiologist.

Eligible participants were adults (≥ 18 years) with PTLD, able to provide written informed consent, with a documented history of smear-positive pulmonary TB (≥ 6 months before enrollment), a negative result for the Xpert MTB/RIF (*Mycobacterium tuberculosis* [MTB] and rifampicin [RIF] resistance) assay (Cepheid) at enrollment, and Medical Research Council (MRC) dyspnea grade ≥ 2.

Participants were excluded if they had comorbidities precluding exercise (eg, unstable cardiovascular disease) or any condition that, in the judgment of the investigator, made participation unsafe.

Participants in the intervention arm received PR in addition to the usual care described below. PR comprised a 6-week program, delivered in groups of 6 to 10 participants, with twice-weekly, 2-hour face-to-face sessions. The exercise regimen was individualized on the basis of baseline ISWT performance and progressively adjusted during the program according to health status and exercise response. Group warm-up and cool-down sessions were provided before and after exercises. Participants then completed 6 exercise stations: 2 for endurance (load-adjustable stationary cycling and walking) and 4 for muscle strengthening (pull-ups, biceps curls, sit-to-stand, and step-ups). Each station involved 3 sets of 8 to 12 repetitions. Participants were also encouraged to continue both endurance and strength exercises at home. Each PR session began with a bespoke group education program covering a range of topics as described in the protocol.^18^ Adherence to the intervention was defined as attending ≥ 75% of PR sessions (9 of 12 face-to-face sessions), and attendance at the 6-week follow-up assessment.

In the absence of PTLD-specific management guidelines, usual care comprised inhalational therapies for spirometry-confirmed airway diseases and verbal advice on smoking cessation and biomass smoke avoidance. All participants underwent frontal chest radiograph and spirometry conducted according to the American Thoracic Society/European Respiratory Society guidelines and the National Health and Nutrition Examination Survey III (NHANES III) reference values.^19^ Participants with spirometry-confirmed medical syndromes were managed according to Global Initiative for Asthma (GINA)^20^ and Global Initiative for Obstructive Lung Disease (GOLD)^21^ recommendations. Before enrollment, participants with medical syndromes were treated until clinically stable to participate in trial activities. All participants were encouraged to stay physically active while at home. Usual care participants were offered PR after the 12-week follow-up visit.

The primary outcome was change in the mean ISWT walking distance, with a minimal clinically important difference (MCID) of 35 m.^22^ The ISWT is an externally paced field test widely used to assess maximal exercise capacity in people with CRD. Exercise prescription is based on data from the ISWT and corresponds to 80% of the maximal performance of the individual on the test.^23^

The secondary outcomes, based on PR minimum measures for low- and middle-income countries,^24^ included respiratory health (Clinical COPD Questionnaire [CCQ] and COPD Assessment Test [CAT] scores), mental health (Hospital Anxiety and Depression Scale [HADS] and Patient Health Questionnaire-9 [PHQ-9]), and functional status (Endurance Incremental Shuttle Walk Test [ESWT], 5-repetition sit-to-stand [FTSTS], and MRC dyspnea scale). Overall health was assessed with the EuroQol Visual Analog Scale (EQ-VAS). All secondary outcomes are responsive to PR in COPD populations with the following MCIDs, respectively: CCQ (0.4),^25^ CAT (2 points),^26^ HADS (2 points),^27^^,^^28^ PHQ-9 (5 points),^29^ ESWT (between 174 and 279 s),^30^ FTSTS (1.7 s),^31^ MRC dyspnea scale (1 point),^32^^,^^33^ and EQ-VAS (8 points).^34^

HRQoL was assessed with the EuroQol (EQ-5D-5L) questionnaire. Using country-specific value sets, responses to the EQ-5D-5L questionnaire were mapped to indices reflecting quality-adjusted life-years (QALYs)^35^^,^^36^ for each observation in the data. As the value sets did not include index values specific to Uganda, we calculated values from all available countries and report the most conservative estimates, based on Zimbabwe index values, in the primary analysis. The change in QALYs from baseline to the 6-week follow-up was then compared between the PR and usual care groups.

The incremental cost-effectiveness ratio (ICER) was calculated as the cost per patient divided by the change in QALYs for the PR, relative to the usual care arm. The calculated ICER was then compared with the cost-effectiveness threshold proposed by the National Institute for Health and Care Excellence (NICE) in the United Kingdom. NICE uses a threshold of £20,000 to £30,000 ($26,000–$39,000/QALY gained)^37^ to determine whether a health care intervention provides good value for money, with interventions below this threshold generally considered cost-effective. Because an equivalent threshold does not currently exist for Uganda, the resulting ICER was adjusted for the purchasing power of Uganda.

Safety was assessed for all randomized participants. In the PR arm, adverse events were considered related if they occurred during exercise and had no other alternative cause.

The study was powered to detect a 35-m difference in the distance walked on the ISWT measured at baseline and after completion of 6 weeks of PR.^22^ Assuming that ISWT follows an approximately normal distribution, a power calculation based on a paired *t*-test was performed. On the basis of a trial sample size of 40 participants in each of the intervention and usual care groups, a 2-sided 5% significance level and a statistical power of 80%, the clinically important change in ISWT of 35 m would also be statistically significant. Our feasibility study was used to obtain an estimate of the pooled SD for the power calculation. Conservatively assuming up to 30% loss to follow-up at 6 weeks, 114 participants were required to be recruited and randomized (1:1) to each arm (57 participants in each group).

All randomized participants were included in the intention-to-treat (ITT) analysis for the primary outcome. Secondary outcomes were analyzed using the modified intention-to-treat (mITT) population, defined as those with ISWT data at baseline and 6-week follow-up.

The primary outcome (change in the mean ISWT distance) was analyzed with a generalized linear mixed model (GLMM), with time point and treatment group as fixed effects. To improve precision, baseline variables that differed by chance between the PR and usual care arms (*P* ≤ .05) were assessed for multicollinearity. The final adjusted model retained CAT score, post-bronchodilator FEV_1_/FVC ratio, and hospitalizations in the preceding 12 months. A mixed-effects GLMM with cluster-specific random intercepts was fitted by maximum likelihood, justified by a high intraclass correlation coefficient (0.72). The goodness-of-fit indices and residual diagnostics indicated good model performance without overdispersion. The results are reported as mean differences with the corresponding 2-sided 95% CI and *P* value < .05 considered statistically significant. The detailed GLMM methodology is provided in the online supplement.

Secondary outcomes were analyzed in the mITT population using a difference-in-differences (DiD) analysis, adjusting for the multiplicity of null hypotheses incorporating a treatment × time interaction. Where relevant, adjustments for multiple comparisons were made using the “mhtexp” package for Stata,^38^ which adjusts *P* values and SEs for correlated outcomes, supplemented by Bonferroni and Holm corrections. SEs were derived from 10,000-iteration bootstraps. Secondary outcomes were additionally evaluated as change scores, using linear regression with cluster-robust SEs. Model diagnostics including histograms, Q-Q plots, and residual-fitted plots showed no major assumption violations.

The full DiD methodology is available in the online article. Continuous variables are presented as mean and SD or as median and interquartile ranges, and categorical data are presented as frequencies and percentages. Normality was assessed to guide use of parametric and nonparametric tests. Group comparisons were done with χ^2^ and Fisher exact tests for categorical data, and *t*-test (normally distributed) and Mann-Whitney *U* test (nonnormally distributed) for continuous data. All analyses were conducted with STATA version 17 software (2021; StataCorp).

The study received ethics approval locally and internationally (MHREC-1478, SS-5105, and University of Leicester, United Kingdom research ethics committee [Ref. No. 22349]), and all participants provided written informed consent before inclusion in the study, in accordance with the Declaration of Helsinki. We followed the Consolidated Standards of Reporting Trials (CONSORT) 2010 reporting guidelines and the CONSORT extension for nonpharmacologic interventions. The trial was prospectively registered at ISRCTN.^39^

## Results

This RCT was conducted from November 20, 2020, to September 30, 2022. Enrollment began on November 20, 2020, but was paused on June 6, 2021, during the second wave of the COVID-19 pandemic due to government restrictions to limit infection spread. The pause increased loss to follow-up because in-person assessments were required per protocol. No participant withdrew, but 22 (19.3%) were lost to follow-up due to COVID-19-related transport disruptions.

**Figure 1:**
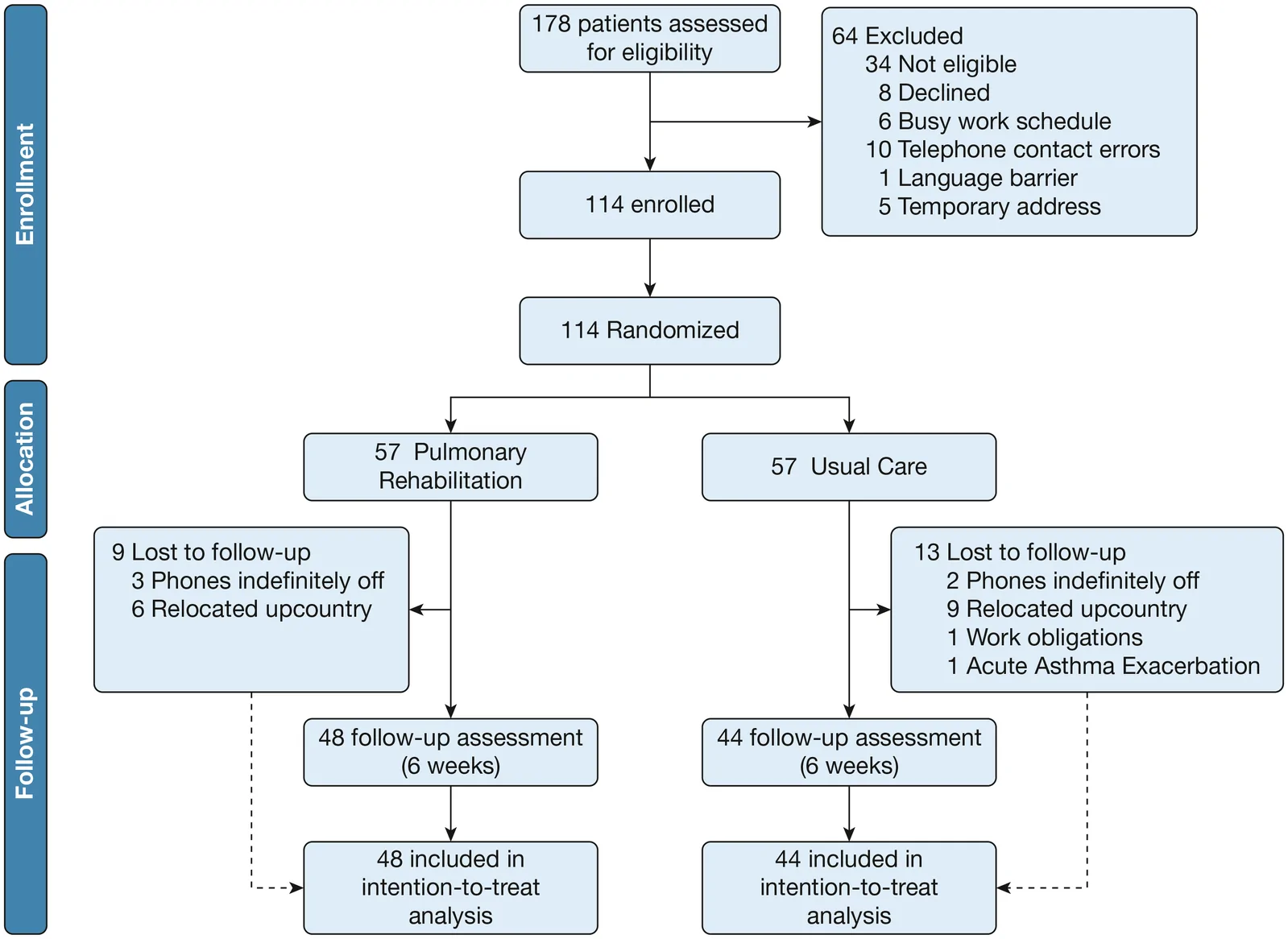
Consolidated Standards of Reporting Trials (CONSORT) diagram of participants in the pulmonary rehabilitation trial for post-TB lung disease.

Of 178 adults with prior PTB treatment screened for study eligibility, 114 (64%) consented and were randomized 1:1 to receive either PR or usual care (Fig 1). Twenty-two participants (9 in PR arm and 13 in usual care arm) missed the 6-week follow-up ISWT.

The baseline demographic and clinical characteristics between the study groups were largely similar (Table 1): mean ± SD age, 43.3 ± 15.2 years; predominantly male (*n* = 65 [57%]). All 114 participants were included in the ITT analysis at 6 weeks follow-up. The mITT analysis included 92 participants (81%) who completed both baseline and 6-week ISWT (48 in PR, 44 in UC). In the PR arm, 41 attended ≥ 75% of face-to-face PR sessions. Twenty-two (19.3%) were lost to follow-up.

**Table 1:** Baseline Characteristics of Participants With Post-TB Lung Disease for the PR Trial, Overall and by Group Allocation

Characteristic	Overall (N = 114)	Pulmonary Rehabilitation (n = 57)	Usual Care (n = 57)
Sex			
Female	49 (43)	23 (40)	26 (46)
Male	65 (57)	34 (60)	31 (54)
Age, y^a^	43.3 (15.2)	45.8 (16.0)	40.8 (14.0)
Age when leaving school, y^a^	18.2 (7.6)	18.1 (9.3)	18.3 (5.3)
Time since completing TB treatment, mo	65.4 (70.5)	66.9 (68.8)	63.8 (72.7)
History of smoking			
Active tobacco smoking	1 (0.9)	1 (1.8)	0 (0)
Previous tobacco smoking	30 (26.3)	16 (28.0)	14 (24.6)
Never smoked tobacco	83 (72.8)	40 (70.2)	43 (75.4)
History of biomass exposure			
Current	43 (37.7)	24 (45.3)	26 (50)
Former	50 (43.9)	6 (11.3)	6 (11.5)
Never	12 (10.5)	23 (43.4)	20 (38.5)
HIV positive	30 (28.9)	16 (31.4)	14 (26.4)
HIV positive while receiving ART	30 (100)	16 (100)	14 (100)
ART duration, mo^a^	119.6 (95.2)	122.9 (108.5)	115.8 (81.3)
BMI, kg/m^2^^a^	20.4 (4.8)	20.5 (5.0)	20.3 (4.6)
Employment status			
Employed in paid work	32 (28.0)	11 (19.3)	21 (36.8)
Self-employed	28 (24.6)	13 (22.8)	15 (26.3)
In unpaid work	2 (1.8)	1 (1.8)	1 (1.8)
Not working	52 (45.6)	32 (56.1)	20 (35.1)
Highest education			
No formal education	2 (1.8)	2 (3.9)	0 (0)
Incomplete primary	21 (18.4)	10 (19.2)	11 (21.6)
Complete primary	14 (12.3)	9 (17.3)	5 (9.8)
Incomplete secondary	37 (32.5)	19 (36.5)	18 (35.3)
Complete secondary	10 (8.8)	4 (7.4)	6 (11.8)
Tertiary	19 (16.7)	8 (15.4)	11 (21.6)
Exercise capacity			
MRC dyspnea grade			
Grade 2	66 (58)	28 (49)	38 (67)
Grade 3	41 (36)	25 (44)	16 (28)
Grade 4	7 (6)	4 (7)	3 (5)
Sit-to-stand 5-s repetitions^a^	9.7 (2.7)	9.9 (3.0)	9.4 (2.4)
Baseline ISWT distance, m^a^	392.6 (131.6)	376.5 (132.0)	408.8 (130)
Endurance Shuttle Walking Test, s^a^	581.7 (362.1)	570.4 (342.1)	592.9 (384.4)
Spirometry			
TB-associated obstruction	29 (25.4)	16 (28.1)	13 (22.8)
Restriction	26 (22.8)	14 (24.6)	12 (21.0)
Reversibility	20 (17.6)	13 (22.8)	7 (12.3)
Normal spirometry	34 (29.8)	12 (21.0)	22 (38.6)
Failed spirometry	5 (4.4)	2 (3.5)	3 (5.3)
Pre-BD FVC % predicted^a^	74.3 (22.5)	69.2 (21.6)	79.5 (22.3)
Pre-BD FEV_1_ % predicted^a^	63.8 (25.1)	57.1 (22.3)	70.5 (26.1)
Respiratory health and HRQoL			
CAT score^a^	17.5 (8.13)	19.7 (7.66)	15.3 (8.06)
PHQ-9 total score^a^	2.01 (3.22)	2.26 (3.2)	1.75 (3.10)
CCQ total score^a^	2.08 (0.89)	2.25 (0.84)	1.91 (0.89)
CCQ symptom score^a^	2.66 (1.19)	2.96 (1.14)	2.37 (1.17)
CCQ mental state score^a^	1.26 (1.37)	1.30 (1.41)	1.22 (1.35)
CCQ functional state score^a^	1.95 (0.90)	2.10 (0.87)	1.80 (0.91)
HADS Depression^a^	1.85 (3.58)	1.81 (3.35)	1.89 (3.83)
HADS Anxiety^a^	1.42 (2.97)	1.19 (2.45)	1.65 (3.43)
EQ-VAS^a^	67.1 (16.2)	64.4 (15.6)	69.7 (16.5)
Comorbidities			
Hypertension	8 (7)	6 (10.5)	2 (3.5)
Cardiac disease	1 (0.9)	1 (1.8)	0 (0)
Arthritis	2 (1.8)	2 (3.5)	0 (0)
Medicines			
LAMA	4 (3.5)	3 (5.3)	1 (1.8)
ICS/LABA	25 (21.9)	16 (28.1)	9 (15.8)
ICS	2 (1.8)	2 (3.5)	0 (0)
Systemic steroids	4 (3.5)	3 (5.3)	1 (1.8)
Cough syrups	7 (6.1)	4 (7.0)	3 (5.3)
Antibiotics	11 (9.7)	6 (10.5)	5 (8.8)
Hospitalizations in the past 12 mo^a^	0.12 (0.32)	0.19 (0.39)	0.05 (0.22)
Nutritional status			
Mid-upper arm circumference^a^	25.7 (22.3)	23.8 (5.3)	27.6 (31.1)
Thigh circumference^a^	45.8 (9.3)	45.8 (8.1)	45.8 (10.4)

Categorical variables are presented as No. (%), unless indicated otherwise. ART = antiretroviral therapy; BD = bronchodilator; CAT = COPD Assessment Test; CCQ = Clinical COPD Questionnaire; EQ-VAS = EuroQol Visual Analog Scale; HADS = Hospital Anxiety and Depression Scale; HRQoL = health-related quality of life; ICS = inhaled corticosteroid; ISWT = Incremental Shuttle Walk Test; LABA = long-acting β-agonist; LAMA = long-acting muscarinic agonist; MRC = Medical Research Council; PHQ-9 = Patient Health Questionnaire-9; PR = pulmonary rehabilitation.

a Values represent mean (SD).

**Figure 2:**
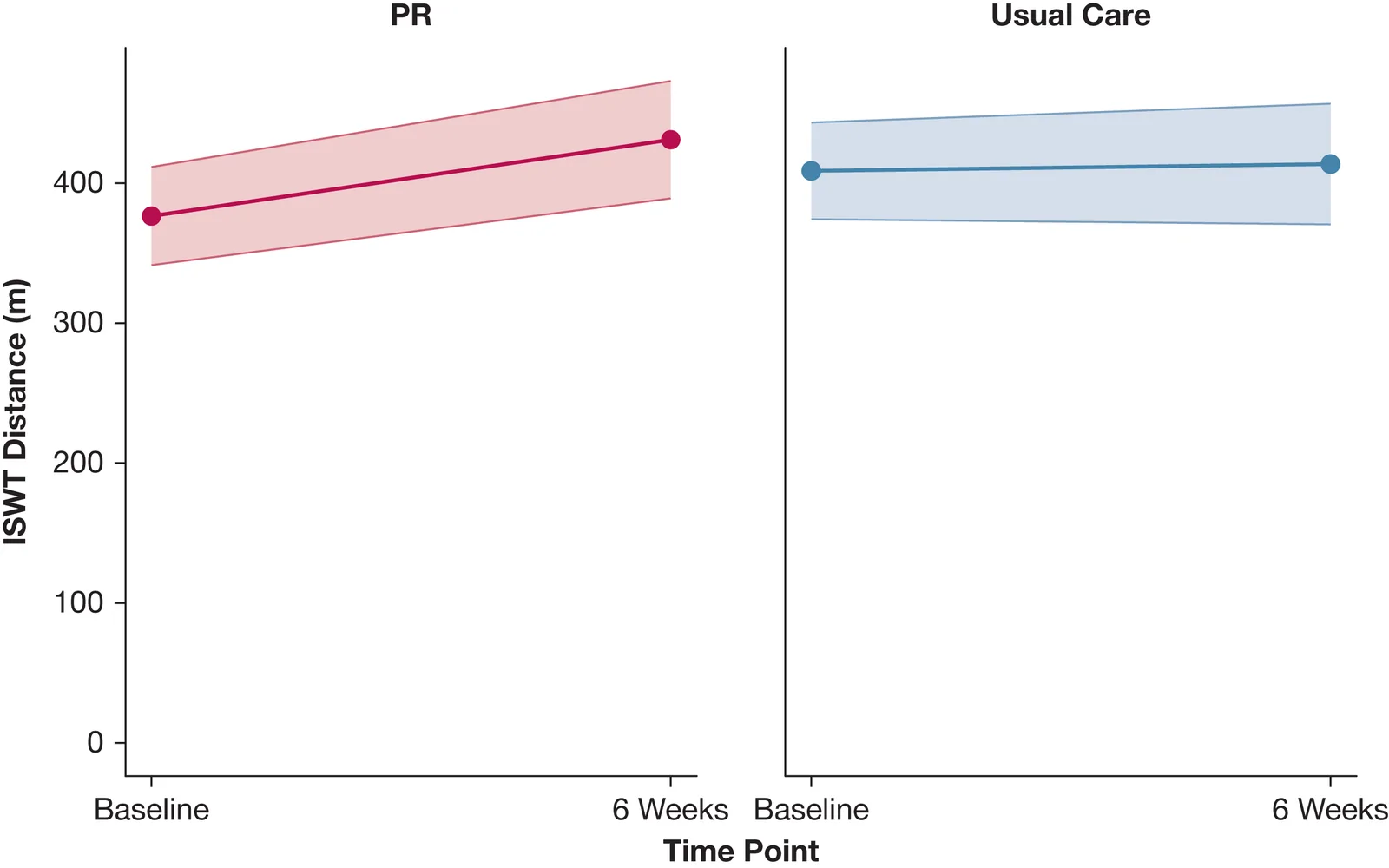
Mean change in ISWT distance between baseline and 6-week follow-up, by group allocation with 95% CIs. ISWT = Incremental Shuttle Walk Test; PR = pulmonary rehabilitation.

The absolute change in ISWT from baseline to 6 weeks was +49.3 m in the PR group and –1.4 m in the UC group, yielding a between-group difference in mean change of 50.7 m. After adjusting for baseline chance imbalances, the GLMM analysis showed that at 6 weeks, the PR group achieved a significantly greater mean ISWT distance and exceeded that in the UC group by 54.36 m (95% CI, 17.22-91.51 m; *P* = .004) (Fig 2). Notably, the mean ISWT improvement in PR exceeded the MCID of 35 m.

The DiD analysis performed on the mITT population for secondary outcomes at the 6-week follow-up (Table 2) showed statistically significant improvements in the PR arm vs usual care arm for the CAT score (–3.6 [95% CI, –6.7 to –0.39]; *P* = .015), CCQ total (–0.37 [95% CI, –0.68 to –0.06]; *P* = .004) and functional domain (–0.40 [95% CI, –0.71 to –0.09]; *P* = .002), as well as MRC dyspnea score (–0.28 [95% CI, –0.54 to –0.2]; *P* = .030). Participants in PR achieved an improvement exceeding the MCID of 2 points for CAT score and reached 0.4 points for CCQ score.

**Table 2:** Adjusted Changes in Study Outcomes From Baseline to 6 Weeks, by Group Allocation

	Pulmonary Rehabilitation Arm			Usual Care Arm			Difference in Difference (95% CI)	*P* Value (Adjusted)
	Baseline Mean (SD)	6 Weeks Mean (SD)	Difference in Means	Baseline Mean (SD)	6 Weeks Mean (SD)	Difference in Means		
Primary Outcome (Intention-to-Treat Population)								
Incremental Shuttle Walk Test, m^a^	381.7 (135.6)	431.0 (144.4)	49.3	415.0 (127.1)	413.6 (141.8)	–1.4	54.36 (17.22 to 91.51)	.004^a^
Secondary Outcomes (Modified Intention-to-Treat Population)^b^								
Endurance Shuttle Walk Test, s	596.1 (360.0)	572.0 (445.8)	–24.1	619.0 (404.8)	613.4 (397.0)	–5.6	–23.7 (–214.2 to 166.9)	.803
MRC dyspnea grade	2.54 (0.62)	1.91 (0.60)	–0.63	2.41 (0.62)	2.02 (0.86)	–0.39	–0.28 (–0.54 to –0.2)	.030
Sit-to-stand repetitions in 5 s	9.91 (3.0)	8.16 (2.3)	–1.75	9.46 (2.33)	8.95 (2.36)	–0.51	–0.67 (–1.65 to 0.32)	.178
CAT score	18.4 (7.9)	10.9 (6.9)	–7.5	14.9 (7.2)	11.2 (8.3)	–3.7	–3.6 (–6.7 to –0.39)	.015
CCQ total	2.12 (0.9)	1.38 (0.8)	–0.74	1.91 (0.84)	1.58 (0.8)	–0.33	–0.37 (–0.68 to –0.06)	.004
CCQ symptoms	2.71 (1.1)	2.01 (1.22)	–0.7	2.36 (1.1)	1.98 (1.2)	–0.38	–0.40 (–0.90 to 0.11)	.036
CCQ functional	1.96 (0.74)	1.34 (0.9)	–0.62	1.82 (0.9)	1.68 (1.0)	–0.14	–0.40 (–0.71 to –0.09)	.002
CCQ mental	1.27 (1.5)	0.19 (0.6)	–1.08	1.2 (1.3)	0.58 (1.1)	–0.62	–0.24 (–0.83 to 0.34)	.416
HADS depression	1.77 (3.1)	0.40 (1.5)	–1.37	2.15 (4.0)	1.0 (2.8)	–1.15	0.15 (–1.25 to 1.55)	.881
HADS anxiety	1.13 (2.2)	0.34 (1.3)	–0.79	1.84 (3.6)	0.91 (2.6)	–0.93	0.37 (–0.64 to 1.38)	.571
PHQ-9	2.34 (3.4)	0.50 (1.5)	–1.84	1.90 (3.3)	1.02 (2.5)	–0.88	–0.5 (–0.73 to 0.73)	.300
BMI, kg/m^2^	20.2 (4.4)	21.2 (4.4)	1.0	20.5 (4.4)	20.9 (4.5)	0.4	0.003 (–0.65 to 0.65)	.993
EuroQol Visual Analog Scale	63.84 (16.1)	79.0 (15.8)	15.16	69.17 (16.7)	79.38 (16.1)	10.21	3.98 (–2.05 to 10.02)	.191

CAT = COPD Assessment Test; CCQ = Clinical COPD Questionnaire; GLMM = generalized linear mixed model; HADS = Hospital Anxiety and Depression Scale; ITT = intention to treat; MRC = Medical Research Council; PHQ-9 = Patient Health Questionnaire-9.

a GLMM analysis of the ITT population.

b Adjustments for multiple comparisons were made using the mhtexp package with *P* values and SEs adjusted for multiple hypotheses.

The DiD analysis of the EQ-VAS group means of the mITT population at 6-week follow-up was positive in the PR arm but not statistically significant (3.98 [95% CI, –2.05 to +10.02]; *P* = .191). After mapping EQ-5D-5L survey responses, the DiD analysis of the resulting QALYs for the mITT population at the 6-week assessment was positive for the PR arm, but not statistically significant (0.02 [95% CI, –0.02 to +0.05]; *P* = .334).

The cost of running the PR program, excluding research expenses, amounted to $6,850, which translates to $114/participant. When evaluated on the basis of the average QALY gained from the PR in the mITT population, this results in a cost of $6,468/QALY gained. After adjusting for the purchasing power of Uganda, the cost was $20,000/QALY gained (GBP £15,400/QALY). However, because the estimated average QALY gained was not statistically significant, the cost-effectiveness should be interpreted with caution, although the results suggest potential value.

The DiD analysis, performed on the mITT population, showed that the improvements were maintained at the 12-week follow-up visit (e-Table 1).

Adverse events (e-Table 2) were reported in 18 (15.8%) trial participants (12 [21%] in the PR arm vs 6 [10.5%] in the usual care arm). The most common adverse event was exacerbation of post-TB airway syndromes (12 of 18; 67%). Two participants in the PR arm experienced severe adverse events requiring hospitalization, both unrelated to the study: 1 due to a road traffic accident post-PR, and another for severe exacerbation of post-TB airway syndrome.

## Discussion

In this RCT of PR in PTLD, participants in the 6-week PR program showed statistically significant and clinically meaningful improvements in exercise capacity and HRQoL, and the intervention was cost-effective. Our study shows that it is possible to set up and deliver a clinical and cost-effective PR program for an underrepresented patient group in a resource-limited setting. This supports the goal of the WHO Rehabilitation 2030 initiative: to make rehabilitation services accessible and affordable, particularly in low- and middle-income countries.^14^

Our findings are consistent with those from a pre-post study of PR in PTLD.^40^ We observed a similar magnitude of improvement in the outcomes of interest, which are corroborated by findings from other observational and underpowered studies in this population.^15^^,^^41^, ^42^, ^43^, ^44^

Our adequately powered clinical trial makes an important contribution to the evidence of PR in the PTLD population. Participants with PTLD registered significant improvements in walking distance, above the group mean MCID of 35 m for a COPD population^22^ we had set for the trial. The change in walking distance observed in our trial was comparable to that seen in other CRDs.^22^^,^^45^^,^^46^ Importantly and interestingly, our study population was much younger and had fewer comorbidities compared with typical COPD cohorts attending PR in higher-income settings.^47^ We had previously established the feasibility of delivering such an intervention to this younger population, and our observed dropout rate was comparable to other PR studies. Our demographic data reveal that many participants traveled significant distances, which in our country took a considerable amount of time. In addition to being younger, our population had a distinct comorbidity profile, with a notable proportion living with HIV. This is rare in high-income countries and seldom reported in PR trials. However, this study was underpowered to assess the impact of HIV on PR for this group. Despite being younger, our cohort (mean age, 43.3 ± 15.2 years) had a baseline ISWT of 392.6 ± 131.6 m, comparable to UK COPD cohorts (483 ± 148 m; mean age, 69.4 ± 9.1 years),^48^ underscoring the impact of PTLD on physical capacity. PR may significantly benefit young, economically active people with PTLD-associated disability.

An additional feature of this trial is the use of the ISWT, a reliable, objective, and more standardized measure of exercise capacity compared with other field walking tests^23^ used in other studies of PR in the PTLD population, described in the systematic review by Alene and colleagues.^15^

Although the symptom burden, HRQoL, anxiety, and depression in our study were lower than in COPD populations,^49^ PR in our PTLD population nonetheless yielded improvements in HRQoL, exercise capacity, and respiratory symptoms. Notably, although the CCQ total score did not reach the MCID, the functional domain did, highlighting the effect of PR on improving physical and social functioning, the key components of HRQoL, and suggesting that domain-specific measures may be more sensitive in capturing therapeutic benefits than aggregate scores. These findings align with the review by Alene and colleagues,^15^ which noted PR benefits, although with caution due to limited data. Anxiety and depression showed marginal improvement, likely due to low baseline levels.

Furthermore, we conducted a cost-benefit assessment, and after adjusting for the purchasing power of Uganda, our PR program was cost-effective in eliciting meaningful clinical improvements, providing a robust foundation of high-quality evidence to support policy change. Our PR program was below the NICE cost-effectiveness threshold of less than £20,000/QALY gained.^50^ Tailored PR programs that address the specific needs of patients with PTLD play a crucial role in improving functional status and quality of life in this population. Providing low-cost PR to patients with PTLD, with no effective treatment options, therefore represents good value for money, considering the health gains achieved relative to the resources required to deliver the program. This could, therefore, impact policy decisions regarding the implementation and provision of PR for the PTLD population, which is often a neglected and underrepresented group of young people in their economically productive years. The evidence from this study supports expanding PR across regions with high numbers of TB survivors and a rising burden of PTLD disability.

The findings from this study should be considered within the context of its limitations. Despite successful randomization, the modest sample size resulted in chance baseline imbalances between groups. These were, however, addressed in the GLMM to improve precision. We chose to consider the value of exercise-based PR in the post-TB treatment phase; however, there may be merit in exploring the value of PR alongside concurrent medical management of TB. In the absence of PTLD-specific outcome measures, we used instruments developed for COPD, which, although fundamentally sensitive, may be limited in capturing experiences and outcomes most relevant to this population, such as stigma. We acknowledge there is no established MCID in PTLD; however, our primary outcome measure was an objective, disease-agnostic measure of performance. In addition, we lacked a country-specific EQ-5D-5L value set for QALY calculation; however, we tested the sensitivity of our analysis by using available sets, all yielding cost/QALY estimates within the NICE threshold. Last, as a single-center RCT, generalizability of these findings may be limited, although they remain informative for similar settings.

## Interpretation

In a young study population with disability associated with PTLD, our 6-week PR program improved exercise capacity in a clinically meaningful manner and was cost-effective.

## Funding/Support

This research was funded by the 10.13039/501100000272National Institute for Health and Care Research (NIHR), UK (17/63/20) using UK aid from the UK government to support global health research. The views expressed in this publication are those of the author(s) and not necessarily those of the NIHR or the UK government. S. J. S. is a NIHR Senior Investigator.

## Financial/Nonfinancial Disclosures

None declared.

## Supplementary Data

e-Online Data

e-Online Data
