# Supplementary material for: Clinical Effectiveness and Cost-Effectiveness of Pulmonary Rehabilitation for People in Uganda With Post-TB Lung Disease: A Randomized Controlled Trial
Source: Chest. 2026 Mar 6;170(1):54–65. doi: 10.1016/j.chest.2025.12.057 (PMC13494459; doi:10.1016/j.chest.2025.12.057)
Supplement: Supplementary file 1 [file mmc1.docx]

**Supplementary material**

**Table of Contents**

[**Methodology used for the GLMM analysis**	2](#_Toc216116560)

[**Methodology used for the difference in difference (DiD) analysis**	2](#_Toc216116561)

[**e-Table 1: Secondary outcomes measured from baseline between intervention and the usual care arm (mITT**	7](#_Toc216116562)

[**e-Table 2*:* Prevalence of A*dverse* E*vent*s and S*erious* A*dverse* E*vent*s by relatedness and severity**	7](#_Toc216116563)

# **Methodology used for the GLMM analysis**

We fitted GLMM to assess the effect of the intervention on the incremental shuttle walk test scores over time. A Gaussian distribution with an identity link was specified for the outcome. Models included fixed effects for group allocation (intervention vs usual care), time (baseline vs follow-up), and their interaction (group x time). To adjust for potential baseline imbalance, all baseline variables that differed between the two study arms (p=<0.05) were considered for inclusion. These were assessed for multicollinearity using Variance Inflation Factors (VIFs) and pairwise correlations. Pre- and post-FEV1% showed severe collinearity (VIF >20; r = 0.97), and CAT and CCQ scores were also strongly correlated (r = 0.82–0.85). To avoid redundancy and ensure model stability, only one representative lung-function measure and one symptom variable were retained. The final adjusted GLMM retained only three baseline variables that did not exhibit collinearity (CAT score, hospitalisations in the prior 12 months, and post-FEV1/FVC ratio.)

Random intercepts were specified at the study level to account for clustering. Models were estimated using maximum likelihood with adaptive Gauss–Hermite quadrature and the identity link. The intra-class correlation coefficient (ICC) was 0.72, indicating substantial between-cluster variability and strong within-study correlation of ISWT outcomes. The high ICC emphasized the need to adjust for cluster-level dependence. Incorporating a random intercept in the GLMM appropriately accounted for this clustering and strengthened the precision and validity of the model estimates and improved predictive accuracy.

The goodness of fit was assessed using various metrics including the log likelihood, the Wald chi^2^ test for fixed effects, and a likelihood ratio (LR) test comparing the mixed-effects model with a standard linear model. The model had a negative log likelihood of -1024.66 and the LR test indicated that including random study-level intercepts significantly improved model fit (LR X^2^ = 59.88, p < 0.001). The residual plots were inspected to check for any patterns that could indicate poor fit. Fixed effects were reported with their estimated coefficients, standard errors, 95% confidence intervals, and p-values. Overdispersion was evaluated by examining model residuals for patterns of increased variability that might indicate poor fit and there was no indication of overdispersion. The residuals were inspected for patterns that would indicate overdispersion, such as increased variability.

# **Methodology used for the difference in difference (DiD) analysis**

The DiD analysis was conducted on multiple outcomes, including changes in exercise capacity, weight, health-related quality of life measures, and respiratory symptom parameters over a 6-week period, with adjustments made for the multiplicity of null hypotheses. The DiD model included interaction terms between treatment (group allocation) and time, comparing the difference in changes between the treatment and control groups for each outcome. A bootstrap method with 10,000 iterations was used to estimate the standard errors, providing robust inference for the DiD estimates. To adjust for the multiplicity of null hypotheses due to multiple outcomes, both Bonferroni and Holm adjustments were applied. These adjustments addressed the increased likelihood of Type I errors that arise when testing multiple outcomes. The p-values obtained from the DiD analysis were adjusted using the Bonferroni and Holm methods, ensuring that the results account for the multiple comparisons made across the different outcomes. Results are reported with the adjusted p-values (Bonferroni and Holm) for each outcome, highlighting the outcomes that remained statistically significant after correction for multiple comparisons.

To check for model diagnostics and robustness, all secondary outcomes were analysed as change scores (baseline to 6 weeks), with treatment effects estimated using linear regression including a fixed effect for group allocation, and cluster standard errors at the participants level to account for heteroscedasticity and within-cluster dependence. For each secondary outcome, we generated, we assessed assumptions by inspecting residual histograms with normal overlays, Q-Q plots and residual vs fitted scatterplots with a fitted line. The diagnostics showed approximately symmetric residuals without systematic curvature. Across outcomes, residual diagnostics were acceptable with approximately normal and pattern-free residuals, wider spread for ESWT and MUAC, consistent with larger room mean squared error (RMSE) and non-significant effects. Inferences were stable across the cluster-robust and 1000 repeated boostrap standard errors and one outcome (thigh circumference) was dropped a priori due to zero variance.

**Below are model diagnostics for the DiD analysis, comprising histograms with normal overlays, Q-Q plots, and residual vs fitted scatterplots for each outcome**

*Change in Incremental Shuttle Walk distance*

*Change in Endurance Shuttle Walk distance*

*Change in MRC dyspnea grade*

*Change in sit-to-stand*

*Change in CAT score*

*Change in CCQ total score*

*Change in CCQ symptom score*

*Change in CCQ functional score*

*Change in CCQ mental score*

*Change in HADS depression score*

*Change in HADS anxiety score*

*Change in PHQ-9 score*

*Change in mid-upper arm circumference*

*Change in Thigh circumference*

*Change in BMI*

*Change in visual analogue scale*

**e-Table 1: Secondary outcomes measured from baseline between intervention and the usual care arm (mITT population)**

# **e-Table 2: Prevalence of Adverse Events and Serious Adverse Events by relatedness and severity**
