# Supplementary material for: Clinical Effectiveness and Cost-Effectiveness of Pulmonary Rehabilitation for People in Uganda With Post-TB Lung Disease: A Randomized Controlled Trial
Source: Chest. 2026 Mar 6;170(1):54–65. doi: 10.1016/j.chest.2025.12.057 (PMC13494459; doi:10.1016/j.chest.2025.12.057)
Supplement: Supplementary file 2 [file mmc2.docx]

**e-Table 1: Between-Group Mean Differences in Secondary Outcomes at 6- and 12-Weeks (mITT population)**

| **Outcome** | **6-week follow-up**  **Difference in group means between PR and Usual care**  **(95% CI)** | ***P*-value** | **12-week follow-up**  **Difference in group means between PR and Usual care**  **(95% CI)** | ***P*-value** |
| --- | --- | --- | --- | --- |
| **Exercise capacity measures** |  |  |  |  |
| Incremental Shuttle Walk Test, (meters) | 50.7 (15.1 to 86.3) | 0.006 | 55.2 (16.4 to 93.9) | 0.006 |
| Endurance Shuttle walking test, (secs) | -23.7 (-214.2 to +166.9) | 0.803 | -92.1 (-270.3 to +86.1) | 0.317 |
| Sit-to-Stand repetitions in 5 seconds | -0.67 (-1.65 to +0.32) | 0.178 | -0.42 (-1.43 to +0.59) | 0.415 |
| MRC dyspnea grade | -0.28 (-0.54 to -0.2) | 0.032 | -0.57 (-0.88 to -0.26) | 0.006 |
| **Health status Measures** |  |  |  |  |
| CCQ total | -0.37 (-0.68 to -0.06) | 0.020 | -0.22 (-0.56 to +0.12) | 0.002 |
| CCQ symptom domain | -0.40 (-0.90 to +0.11) | 0.122 | -0.20 (-0.69 to +0.28) | 0.412 |
| CCQ functional domain | -0.40 (-0.71 to -0.09) | 0.013 | -0.26 (-0.65 to +0.12) | 0.172 |
| CCQ mental domain | -0.24 (-0.83 to +0.34) | 0.821 | -0.17 (-0.75 to +0.42) | 0.581 |
| HADS Depression | 0.15 (-1.25 to +1.55) | 0.832 | 0.72 (-0.79 to +2.23) | 0.354 |
| HADS Anxiety | 0.37 (-0.64 to +1.38) | 0.474 | 0.87 (-0.29 to +2.04) | 0.144 |
| PHQ-9 depression score | -0.5 (-0.73 to +0.73) | 0.421 | -0.41 (-1.68 to +0.85) | 0.523 |
| CAT Score | -3.6 (-6.7 to -0.39) | 0.028 | -5.51 (-8.30 to -2.73) | 0.002 |
| EuroQol VAS | 4.95 (-1.64 to +11.54) | 0.191 | 7.1 (0.13 to 14.0 | 0.046 |
| **Nutritional status** |  |  |  |  |
| BMI (Kg/M^2^) | 0.003 (-0.65 to +0.65) | 0.993 | 0.44 (-0.44 to +1.32) | 0.433 |
| Mid Upper Arm Circumference, (cm) | 5.40 (-4.81 to +15.6) | 0.304 | 5.61 (-5.53 to +16.76) | 0.324 |
| Thigh Circumference, (cm) | 0 | .. | -0.58 (-3.00 to +1.85) | 0.635 |

**Legend:** MRC: Medical Research Council, CCQ: Clinical COPD Questionnaire, HADS: Hospital Anxiety and Depression Scale, PHQ: Patient Health Questionnaire, CAT: COPD assessment Test, EuroQol-VAS: EuroQol Visual Analogue Scale, BMI: Body Mass Index

**Adverse Events and Safety**

**e-Table 2: Adverse Events and Serious Adverse Events by Relatedness and Severity**

| **Baseline characteristics** | **Pulmonary Rehabilitation** | **Usual Care** | **Total** |
| --- | --- | --- | --- |
| **Total AEs** | **10** | **8** | **18** |
| **By severity**  *Mild*  *Moderate*  *Severe* | 6  2  2 | 7  1  0 | 13  3  2 |
| **Causality**  *Certain*  *Probable*  *Possible*  *Conditional*  *Assessable* | 0  0  7  3  0 | 0  0  0  8  0 | 0  0  7  11  0 |
| **AEs within 6 weeks** | **8** | **5** | **13** |
| **AEs within 12 weeks** | **2** | **3** | **5** |
|  | | | |
| **Total SAEs** | **2** | **0** | **2** |
| **Criteria**  *Death*  *Life threatening*  *Hospitalization*  *Medically significant*  *Not applicable* | 0  0  2  0  0 | 0  0  0  0  0 | 0  0  2  0  0 |
| **By severity**  *Mild*  *Moderate*  *Severe* | 0  1  1 | 0  0  0 | 0  1  1 |
| **Causality**  *Related*  *Probably related*  *Possibly related*  *Unlikely related*  *Unrelated* | 0  0  0  0  2 | 0  0  0  0  0 | 0  1  0  1  0 |
